# Clinical Characteristics and Risk Factors for Severe Dengue Fever in Xishuangbanna, During the Dengue Outbreak in 2019

**DOI:** 10.3389/fmicb.2022.739970

**Published:** 2022-03-10

**Authors:** Xiaodan Wang, Tingting Li, Yun Shu, Juan Zhang, Xiyun Shan, Daiying Li, Dehong Ma, Shuying Long, Yue Pan, Junying Chen, Pinghua Liu, Qiangming Sun

**Affiliations:** ^1^Institute of Medical Biology, Chinese Academy of Medical Sciences, Peking Union Medical College, Kunming, China; ^2^Yunnan Key Laboratory of Vaccine Research & Development on Severe Infectious Diseases, Kunming, China; ^3^Yunnan Key Laboratory of Vector-Borne Infectious Disease, Kunming, China; ^4^Xishuangbanna Dai Autonomous Prefecture People’s Hospital, Jinhong, China; ^5^Kunming Medical University, Kunming, China

**Keywords:** severe dengue fever, IgG, IgM, dengue inpatients, dengue gene sequence

## Abstract

**Background:**

Dengue poses a large burden on the public health systems worldwide. severe dengue (SD) could lead to more serious clinical symptoms and even death. This study aimed to identify the cause of SD in a clinical trial during the dengue outbreak in Xishuangbanna in 2019, and could provide new insights into the pathogenic mechanisms of SD.

**Methods:**

Mosquito-borne viral (DENV, JEV, and CHIKV) infections were identified. The epidemiological factors and clinical symptoms of inpatients in Xishuangbanna were recorded. The IgG and IgM levels in the serum of dengue inpatients were evaluated, and secondary infections were identified. Then, the structural proteins (C/PrM/E) were sequenced and compared with those of the same type of DENV in the same area as before, and their structures were predicted by the SWISS-MODEL (expasy.org). The full-length viral genomes were sequenced and aligned with representative strains by BioEidt or MEGA 5.0.

**Results:**

In this outbreak, the clinical symptoms were more serious in SD. The proportion of SD inpatients of male and Han nationality was larger than that of dengue fever (DF) inpatients (*p* < 0.05). DENV-2 infection was the majority in DF, with 45 inpatients. However, DENV-1 infection was the most common SD, with 54 inpatients. There were 3 DENV-3-positive inpatients in the DF group and 6 ZIKV-positive inpatients in the SD group. A secondary infection accounted for 76.47% (78 cases) of SD inpatients, but secondary infections were only in 20% (17 cases) of DF inpatients. In the three-dimensional structure of protein analysis, the C/PrM/E of DENV-1 and DENV-2 showed more stability than previous epidemic strains, while DENV-3 in 2019 showed a looser spatial structure. After a complete genome sequencing and analysis, all six DENV-2 strains belonged to cosmopolitan, five of which clustered into one branch. The GC/AT of the five strains decreased from 2014 to 2018. Compared with DF strains, SD strains had no mutations of commonness.

**Conclusions:**

SD may related to secondary heteromorphic dengue in Xishuangbanna in 2019. The coinfection of ZIKV could be another related factor for SD. The currently datas were very limited and only suggestive.

## Introduction

Dengue virus (DENV) is a mosquito-borne flavivirus that is transmitted by the vectors, *Aedes aegypti* and *Aedes albopictus*, and is a vector-borne infectious disease virus (Hawley et al., 1987). Dengue virus is a single stranded, positive RNA virus with an envelope genome of approximately 11 kb. The genome encodes a polyprotein, which is processed into three structural proteins [the capsid (C), premembrane (prM), and envelope (E) protein] and seven non-structural proteins (NS1-NS5) (Guzman and Harris, 2015). There are currently four circulating serotypes (DENV-1 to DENV-4) that exhibit up to 70% sequence homology (Bhatt et al., 2020). The incubation period of dengue virus infection is 4–7 days (Bhatt et al., 2020). The disease spectrum ranges from asymptomatic infection and moderate febrile illness (DF) to more severe dengue (SD), such as dengue hemorrhagic fever (DHF) and dengue shock syndrome (DSS; Chaturvedi et al., 2000). The clinical symptoms of SD patients mainly include high fever, severe pain in the bones, joints and muscles, headache, skin rash, lymph node enlargement, bleeding, shock and even death (World Health Organization, 2009).

**FIGURE 1:**
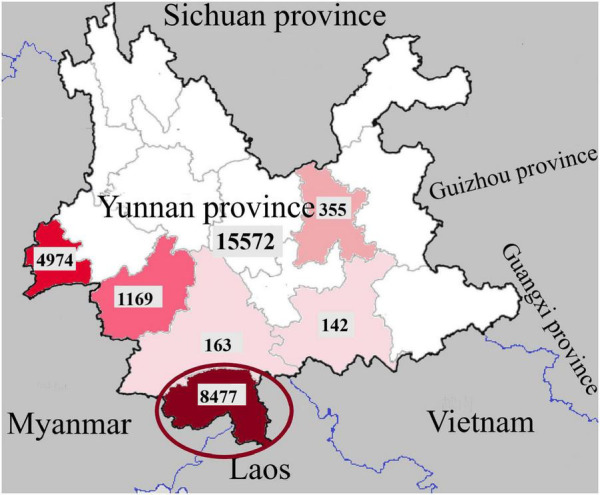
Regional distribution of dengue fever cases in Yunnan Province, China, 2013-2019 (the dates in the picture are from Zhang, 2021).

Dengue was listed as a potential threat among ten diseases by the WHO in 2019 (Norshidah et al., 2021). The global incidence has been estimated at 390 million infected individuals each year. In China, no case was reported from 1949 to 1977 until an outbreak occurred in Guangdong Province in 1978 (Yue et al., 2020). In recent years, dengue cases have been reported in almost all provinces (autonomous regions) in China (Liu, 2020). Southeast Asia is an important area for *Aedes aegypti* and *Aedes albopictus* and has always been the main epidemic area of dengue disease. Yunnan, as one of the border provinces of China, is adjacent to the Southeast Asian countries Laos, Myanmar and Vietnam. A total of 15,572 dengue cases were recorded in Yunnan Province from 2013 to 2019, as shown in Figure 1. Dengue cases were concentrated in the border areas, and a total of 8,477 dengue cases were recorded in Xishuangbanna Prefecture (red circle in Figure 1), bordering Laos and Myanmar, including 568 imported cases (6.70%) and 7,909 local cases (93.30%) (Zhang, 2021). In Xishuangbanna, few cases of dengue virus infection were reported before 2013. The number of reported dengue virus infections (DENV-3) rose to 1,319 in 2013, 1,132 in 2015 (DENV-2) and 1348 in 2017 (DENV-1). As of November 2019, the number of dengue virus NS1 positive infections exceeded 3,900 (Zhang et al., 2021). With the increase in the number of infections, the number of inpatients with SD increased to 102 in 2019. According to previous reports, 70 of 634 inpatients (11.04%) had SD in 2013 (Ma et al., 2016). Among the 109 inpatients in 2015, 13 (11.9%) had SD (Cui et al., 2016).

As a more serious form of dengue infection, SD is directly life-threatening. In some areas, the mortality rate of pediatric patients is as high as 5% (Wang et al., 2016). Former studies suggest that age, gender, social status, genetic background, chronic diseases might adversely influence the clinical presentation of dengue infection (Htun et al., 2015). The aim of this article is to study the factors associated with SD. The infection of mosquito-borne viruses [including Zika virus (ZIKV), Japanese encephalitis virus (JEV), and Chikungunya virus (CHIV)] and the serotype of DENV were identified, and the epidemiological factors and clinical symptoms of inpatients in Xishuangbanna were recorded. Then, IgG and IgM in the serum of dengue inpatients were detected, and secondary infections were identified. The structural proteins (C-PrM-E) were sequenced and compared with those of the same type of DENV in the same region. The three-dimensional structure of dengue virus structural proteins was predicted by the SWISS-MODEL (expasy.org). Finally, whole genome sequences of 6 inpatients (including 3 SD and 3 DF) were obtained and compared with the sequences of different viruses from different years to detect the homology of the sequence.

## Materials and Methods

### Study Design and Participants

**FIGURE 2:**
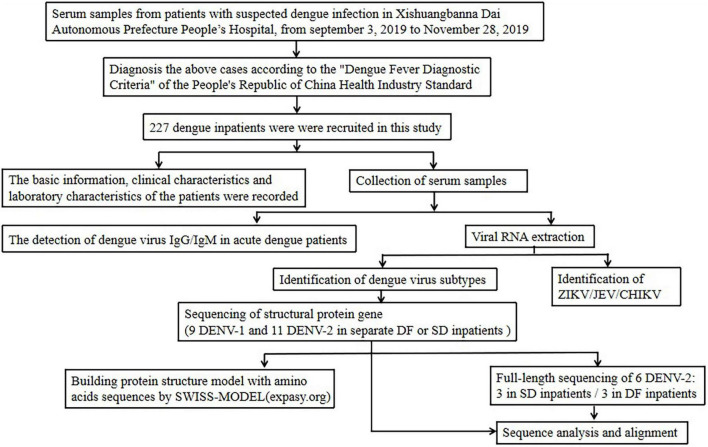
Study design and participants.

Laboratory-confirmed dengue fever inpatients admitted to the People’s Hospital of Dai Autonomous Prefecture of Xishuangbanna from September to November 2019 were enrolled in this study. Patients were diagnosed based on the Guidelines for the Diagnosis, Treatment, Prevention and Control of Dengue Fever (World Health Organization, 2009). Data on clinical symptoms and laboratory tests were collected for the analysis. Laboratory test data included the white blood cell count (WBC) and the platelet count (PLT). The overall study design is shown in Figure 2.

### Mosquito-Borne Virus Identification

A total of 225 DENV-positive serum samples of inpatients were collected from inpatients in the People’s Hospital of Dai Autonomous Prefecture of Xishuangbanna. Dengue NS1 antigen was detected using a DF NS1 test kit (Blue Cross, Beijing, China). Viral RNA was extracted from 140 μL of serum using a QIAamp Viral RNA Mini Kit (QIAGEN, Hilden, Germany) according to the manufacturer’s instructions and stored at −80°C. Viral RNA was used for PCR the identification of the dengue viral subtypes, Zika virus (ZIKV), Japanese encephalitis virus (JEV) and Chikungunya virus (CHIKV), following the identification of flavivirus. The primers were as Supplementary Table 1 shown (Wang et al., 2016). All dengue virus PCR positive samples were labeled for the next step, which included the amplification of the whole gene or the structural protein nucleic acid sequence.

### IgG and IgM Antibodies of DENV Detection

DENV IgG and IgM antibodies were detected by enzyme-linked immunosorbent assay (ELISA) (Order Nr: IB05044 or IB05045, Immuno-Biological Laboratories, Inc., Minneapolis, MN, United States) in 225 DENV-positive serum samples of inpatients, according to the instructions of the manufacturer.

### Determination of Primary and Secondary Dengue Virus Infection

The judgment basis of primary and secondary infection was defined as follows (Wei et al., 2016): for specimens taken less than 7 days after the onset, both IgM and IgG antibodies were negative, the judgment could not be made. If the IgM antibody was positive, IgG antibody was negative, the judgment was primary infection. The judgment of secondary infection is that both antibodies are positive, or if IgM antibody was negative, IgG antibody and the DENV RNA are both positive.

### Analysis of the Amino Acid Sequence of DENV Structural Proteins (C-PrM-E)

Twenty DENV nucleic acid-positive samples were randomly selected to sequence the nucleic acid sequence of DENV structural proteins and then translated into amino acids with BioEdit 7.0. The C-PrM-E structure was used to build a protein structure model with amino acid sequences by the SWISS-MODEL (expasy.org).

### Amplification of the Full-Length Genome and Analysis of Isolated DENV Nucleotides

Serum from 3 DF inpatients and 3 SD inpatients was selected for sequencing the full-length genome of DENV, all the six samples were from the same twenty DENV nucleic acid-positive samples used. The primers were used in this study were from our former study (Jiang et al., 2018). Sequences were analyzed using BioEdit 7.0 and compared with sequences available from the BLAST database (blast.ncbi.nlm.nih.gov/Blast.cgi). Phylogenetic analyses were performed using the neighbor-joining method with the Tajima-Nei model (MEGA, version 6.0^1^). The DENV genotype was analyzed using the related reference sequences in NCBI (National Center for Biotechnology Information, Minneapolis, MN, United States) and with known genotypes in the phylogenetic tree. The information of reference sequences were shown in Supplementary Table 2.

### Statistical Analysis

The continuous variables were described by the mean ± standard deviation, and the categorical variables were described by the constituent ratio. Differences or associations with *p*-values <0.05 were considered significant. All data analyses were performed using SPSS 22.0 software (IBM, Armonk, NY, United States) and GraphPad Prism 7.

### Ethical Approval

Institutional Review Board approval was obtained from the Ethics Committee of the Institute of Medical Biology, Chinese academy of Medical Sciences, China. All procedures that were performed in the studies involving human participants were in accordance with the ethical standards of the institutional and/or national research committee and with the 1964 Helsinki declaration and its later amendments or comparable ethical standards.

## Results

### Basic Characteristics of Dengue Inpatients

In this study, there were 102 SD patients among the 225 dengue inpatients. Compared with the general dengue patients, the proportions of male and Han nationality inpatients with SD were larger (*p* < 0.05) (Table 1). The mean age of DF inpatients was 48.67, the median was 49 (2–97). The youngest is 2 years old and the oldest is 92 years old in DF. The mean age of SD inpatients was 46.14, and the median was 43 (13–88). The youngest is 13 years old and the oldest is 88 years old in SD. The ratio of males to females in SD was 1.76, which was higher than that in DF (0.81). The clinical symptoms were more serious in SD. Compared with DF, the number of low platelet counts (PLT) in SD was greater than that in dengue patients (*p* < 0.01). Unexpectedly, other clinical sympt**o**ms, including fever, vomiting, muscle pain, bleeding, coma, convulsions, and white blood cell counts (WBT), between DF and DHF were no significant differences.

**TABLE 1:** Comparison of characteristics of moderate and severe dengue fever inpatients.

Characteristics	DF inpatient	SD inpatient	*X* ^2^	*P*-value
**Basic characteristics of dengue patients**				
Male	47	65	7.483	0.006
Female	58	37		
**Age**				
Average age	48.67	46.14	–	–
Median Age (Min-Max)	49 (2–97)	43 (13–88)	–	–
Minorities (Permanent population)	40 (36)	10 (7)	22.61	≤0.01
The Han nationality (Permanent population)	65 (48)	92 (70)		
**Underlying diseases in dengue patients**				
Diabetes	13 (10.57%)	10 (9.80%)	0.36	0.85
Hypertension	13 (10.57%)	16 (15.69%)	1.30	0.25
Diabetes and hypertension	8 (6.50%)	3 (2.94%)	0.85	0.36
**Clinical symptoms**				
Fever (≤39°C)	95	72	0.64	0.42
Fever (>39°C)	21	21		
Vomiting	11	17	1.70	0.19
Muscle pain	68	63	0.20	0.66
Bleeding	6	13	3.07	0.08
Coma	4	2	0.65	0.42
Convulsions	4	1	1.79	0.18
WBT/10^9^.L^–1^	118	101		
>10	4 (3.34%)	3 (2.97%)	–	–
4∼10	48 (40.67%)	31 (30.69%)	2.35	0.13
<4	66 (55.93%)	67 (66.34%)	2.470	0.12
PLT/10^9^.L^–1^	117	100		
>100	62 (52.99%)	16 (16.00%)	32.04	<0.01
30∼100	47 (40.17%)	21 (21.00%)	9.21	<0.01
<30	8 (6.84%)	63 (63.00%)	77.26	<0.01
Total inpatients	123	102	–	–

A total of 23 diabetics and 29 hypertension patients were found in 225 dengue inpatients. There were 11 inpatients suffering from both two diseases, and 3 of them are SD inpatients. As Table 1 shown, in DF inpatients, 10.57% of patient have diabetes or hypertension, 4.89% have both two diseases. As for SD inpatients, 9.80% of patient have diabetes, 15.69% have hypertension, and only 2.94% have both two diseases. There is no statistical difference between the two groups.

### Mosquito-Borne Virus or Virus Coinfection Identification

After nucleic acid samples were extracted and evaluated with specific primers for the presence of different viruses (DENV 1-4, ZIKV, CHIKV, JEV), 54 DENV-1 and 22 DENV-2 were found in 102 samples of sera of SD inpatients. There was one coinfected DENV-1 patient with ZIKV and five coinfected DENV-2 patients with ZIKV. All other viruses were negative. Similarly, among 122 common DF inpatients, there were 19 DENV-1, 45 DENV-2 and 3 DENV-3. The main epidemic serotype of DF inpatients was DENV-2, but the main serotype of SD patients was DENV-1, as shown in Table 2.

**TABLE 2:** Mosquito-borne virus (DENV 1-4, ZIKV, CHIKV, JEV) or virus coinfection identification.

	DENV-1	DENV-2		DENV-3	DENV-4	ZIKV	CHIKV	JEV
DF inpatient	19	45		3	0	0	0	0
SD inpatient	54	22		0	0	6	0	0
X^2^	23.82	–				–	–	–
*P*-value	*P* < 0.01	–				–	–	–

### Determination of Primary and Secondary Dengue Virus Infection

IgG and IgM were detected in 85 DF inpatients, of which 44 were IgM positive and 19 were IgG positive, as shown in Table 3. In all IgG-positive inpatients, the onset time was less than or equal to 7 days in 17 cases and more than 7 days in 2 cases. IgG and IgM were detected in 95 patients with SD. Among them, 90 patients were IgM positive, and 84 patients were IgG positive. Among all the IgG-positive patients, 78 had an onset time less than or equal to 7 days. According to the judgment basis of primary and secondary infections as previously described in the methods, 78 were secondary infections in SD inpatients and 17 were secondary infections in DF inpatients.

**TABLE 3:** IgG and IgM antibodies for DENV detection in dengue inpatients.

		DF inpatients		SD inpatients		*X* ^2^	*P*-value
			
		IgM	IgG	IgM	IgG		
≤7	Positive	40	17	85	78	46.35^a^	≤0.01
	Negative	41	76	4	11	107.79^b^	
>7	Positive	4	2	5	6	—	—
	Negative	0	0	1	0	—	—
Total		85	85	95	95	—	—

*^a^, compared for IgM. ^b^, compared for IgG.*

### Analysis of the Amino Acid Sequence of DENV Structural Proteins (C/PrM/E)

The amino acid sequences of DENV-1, DENV-2 and DENV-3 structural proteins (C/prM/E) were compared by BioEdit. In DENV-1s, compared with the strain KY672931.1 (2015), there were three amino acid mutations of the C protein in strain 5 (2019), from proline(P) to Serine (S), Arginine (R) to Lysine (K) and K to R. Two amino acid mutations were observed in the E protein, including a Leucine (L) to Isoleucine (I), Valine (V) to Alanine (A), and but there were no mutations in the PrM protein. In DENV-2s, compared with strain KY672955.1 (2015), there was one amino acid mutation in the C protein in 15 (2019), K to R. Two amino acid mutations in the PrM protein included K to R, V to A, and one amino acid (L to I) mutation in the E protein. In DENV-3s, compared with strain KR296743.1 (2013), there were two amino acid mutations in the C protein, K to R, asparagine (N) to I, five amino acid mutations in the PrM protein, and ten amino acid mutations in the E protein.

The possible three-dimensional structures of the structural proteins of DENV-1, DENV-2 and DENV-3 in 2019 were later predicted and compared with those of the same type of DENV in Xishuangbanna Prefecture as previously (Table 4). Homology modeling revealed that four strains of DENV-1/DENV-2 had the same three-dimensional structure. However, the two strains of DENV-3 were different. Among the 21 mutation sites in C/prM/E, there were 11 hydrophobic amino acids and 10 hydrophilic amino acids in 2013. However, there were 8 hydrophobic amino acids and 13 hydrophilic amino acids in 2019. The decrease in hydrophobic amino acids in 2019 led to a looser structure than the strain in 2013.

**TABLE 4:** The amino acid sequences of DENV-1, DENV-2 and DENV-3 structural proteins (C/prM/E) were compared by BioEdit, and the protein structure was predicted by SWISS-MODEL (expasy.org).

DV1	C				PrM								E										
	27	99	100		–								128				396						
2015	P	R	K		–								L				V						
2019	S	K	R		–								I				A						

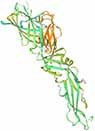													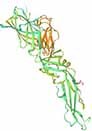										
China Yunnan 2015 (KY672931.1)													5 2019										

**DV2**	**C**			**PrM**									**E**										

	100			165				166					20										
2015	K			K				V					L										
2019	R			R				A					I										

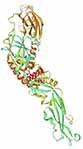													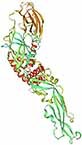										
China Yunnan 2015 (KY672955.1)													15 2019										

**DV3**	**C**		**PrM**						**E**														

	86	90	8	12	40	69	71		14	95	138	246	154	168	183	185		315	394	397	461	466	503
2013	K	N	M	T	I	L	A		A	I	S	H	T	D	V	S		L	I	K	G	I	T
2019	R	I	I	A	T	H	T		T	V	L	Y	I	E	T	T		T	T	N	S	V	A

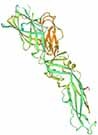													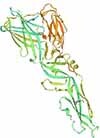										
China Yunnan 2013 (KR296743.1)													5 2019										

																							

### Phylogenetic Analysis of Isolated DENV Nucleotide

To analyze whether the sequence is a factor for the severity of dengue patients, we randomly selected three DENV-2 strains from each of the severe and mild patients for whole genome sequencing. After sequencing, nucleic acid and amino acid sequences were analyzed.

**FIGURE 3:**
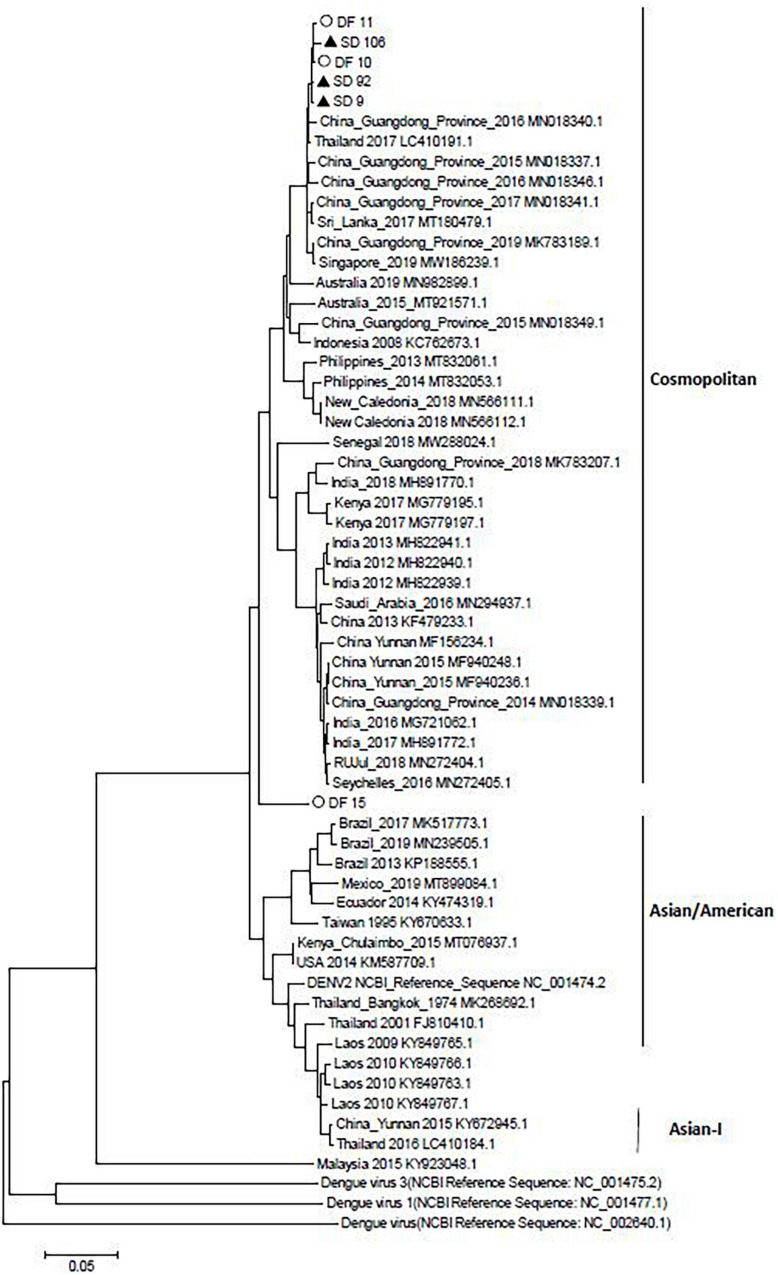
Neighbor-joining phylogenetic tree generated using the nucleotide sequences of complete dengue virus sequences. Study sequences are labeled in black triangles or hollow circles. Others are standard sequences, including sequences of the DENV-2 subgenotype retrieved from the NCBI GenBank. Phylogenetic trees were constructed by the neighbor-joining method and the Kimura 2-parameter model by the MEGA package.

In the nucleotide composition analysis, the sequences from DF inpatients (DF10, DF11, and DF15) or SD inpatients (SD9, SD92, and SD106) were compared with other sequences in China from different years, including the strains, MF940237.1 (China Yunnan Province 2015), MN018339.1 (China Guangdong Province 2014), MN018337.1 (China Guangdong Province 2015), MN018340.1 (China Guangdong Province 2016), MN018341.1 (China Guangdong Province 2017), MK783207.1 (China Guangdong Province 2018), and the DENV-2 standard strain NCBI Reference Sequence (NC 001474.2). The results showed that the GC/AT of DENV-2 in China decreased from 2014 to 2018, except the MN018341.1 strain (China Guangdong Province 2017) (Table 5). However, the GC/AT in five of six strains increased in 2019, and the portion rose to the level of 2014. Compared with the other five strains, the GC/AT of DF15 was closer to that of MK783207.1 (China Guangdong Province 2018). Compared with DF strains, SD strains had no mutations of commonness. Although DF15 is different from other virus strains, an evolutionary tree analysis showed that it belongs to the cosmopolitan type, similar to the other five viruses. According to the phylogenetic analysis, DENV-2 of the 2019 dengue outbreak in Yunnan most likely originated from the China Guangdong Province or Thailand, not Yunan Province (Figure 3).

**TABLE 5:** Basic information on the DENV-2 sequences was analyzed by BioEdit.

Viral strain	A	C	G	T	G + C	A + T
NC_001474.2	3375 (33.17%)	2079 (20.43%)	2574 (25.29%)	2148 (21.11%)	45.73%	54.27%
MF940237.1	3367 (33.09%)	2084 (20.48%)	2581 (25.36%)	2144 (21.07%)	45.84%	54.16%
MN018339.1	3363 (33.05%)	2082 (20.46%)	2585 (25.40%)	2146 (21.09%)	45.86%	54.14%
MN018337.1	3377 (33.19%)	2083 (20.47%)	2573 (25.28%)	2143 (21.06%)	45.75%	54.25%
MN018340.1	3367 (33.09%)	2080 (20.44%)	2569 (25.25%)	2160 (21.23%)	45.69%	54.31%
MN018341.1	3364 (33.06%)	2071 (20.35%)	2581 (25.36%)	2160 (21.23%)	45.72%	54.28%
MK783207.1	3370 (33.11%)	2066 (20.30%)	2580 (25.35%)	2163 (21.25%)	45.64%	54.36%
DF11	3367 (33.09%)	2092 (20.56%)	2576 (25.31%)	2135 (20.98%)	45.87%	54.07%
DF15	3383 (33.24%)	2083 (20.47%)	2562 (25.18%)	2147 (21.10%)	45.65%	54.34%
DFF11	3368 (33.10%)	2093 (20.57%)	2578 (25.33%)	2136 (20.99%)	45.90%	54.09%
SD9	3372 (33.14%)	2089 (20.53%)	2574 (25.29%)	2141 (21.04%)	45.82%	54.18%
SD92	3368 (33.10%)	2089 (20.53%)	2577 (25.32%)	2142 (21.05%)	45.85%	54.15%
SD106	3367 (33.09%)	2086 (20.50%)	2578 (25.33%)	2145 (21.08%)	45.83%	54.17%

## Discussion

Dengue poses a large burden on the public health systems worldwide. Due to the lack of an ideal animal model, the pathogenesis of dengue has not yet been elucidated. SD is currently believed to be mainly related to secondary heteromorphic DENV infection, coinfection of mosquito-borne viruses, viral variation and host immune response (Simmons et al., 2012; St. John et al., 2013). The purpose of this study was to investigate the clinical characteristics and risk factors for severe dengue fever in Xishuangbanna, during the dengue outbreak in 2019.

As a more serious consequence of dengue, SD patients often have more serious clinical symptoms. However, in this outbreak, the only significant difference between SD and dengue patients is the platelet counts. The number of low platelet counts in SD was greater than that in dengue patients (*p* < 0.01). But there were no significant differences between DF and DHF in other clinical symptoms. The small sample size was the main reason for those results and the basic characteristics of certain groups were also very important for the pathogenesis of SD. In South America, Southeast Asia and other countries, SD is considered to occur in children and infants (Sharp et al., 2017). However, in this study, there were only two SD inpatients (13 and 18 years old) younger than 18 years old. The mean ages of SD and DF were 46.14 and 48.67, respectively, which were not significantly different. Compared with DF inpatients, SD inpatients were more likely to be male and of Han nationality (*p* < 0.05). The formation of SD is not related to age but is related to gender. The reason may be that the spread of dengue is related to the population mobility. Compared with females, males have a larger proportion of migrant workers and are more vulnerable to mosquito bites, which are more likely to lead to SD.

In 2019, there were 22599 cases of dengue fever, with an incidence rate of 1.63/10 million (Liu, 2020). As a typical dengue epidemic area, in 2019, the main epidemic type in Xishuangbanna Prefecture was DENV-1, with an incidence rate of up to 67%. DENV-2 accounted for 32%, and only one patient had DENV-3, which was consistent with our assumption that the epidemic trend was dengue virus. Although DENV-1 was prevalent in Xishuangbanna in 2017, DENV-1 was still prevalent in Xishuangbanna in 2019. According to former studies, DENV-2 and DENV-3 are more likely to cause SD than DENV-1 and DENV-4 (Fried et al., 2010). Among the first infections of this outbreak, 6 SD inpatients were infected with DENV-2, and 3 SD inpatients were infected with DENV-1. After infection with one DENV serotype for the first time, the serogroup cross reactive antibody produced by the host usually can only protect from other serotypes of DENV infection for 3–6 months. When the host is reinfected with heterotypic DENV, the E or PrM antibody produced by the first infection causes a subneutralization titer in the body and forms an immune complex with the virus being infected, increasing the infection rate and replication of the virus (Murphy and Whitehead, 2011). Therefore, we compared the nucleic acid sequence of the PrM protein of the DENV strain in 2019 with that of the dengue virus strain in the same area. Compared with the previous same virus strain genotype, little difference was observed in the primary structure of DENV-1 and DENV-2, but the higher structures were the same. DENV-3 had some differences in the primary structure, which led to different higher structures. These results indicated that DENV-1 and DENV-2 may be more stable than DENV-3. Previous epidemiological studies have shown that the outbreak of DENV-3 and DENV-2 occurred earlier than that of DENV-1 and was more able to lead to subneutralization titers in first infected people. Thus, DENV-1 should be the main serotype in subsequent secondary infections. Among the secondary infection SD inpatients in this study, 50 (%) were DENV-1 and 13 (%) were DENV-2, which was consistent with our study.

ZIKV, CHKIV, and JEV are also transmitted by the mosquito. When there were multiple arboviruses in one place at the same time, humans may be infected with different types of arboviruses at the same time through mosquito bites. In this study, we compared the coinfection of ZIKV, CHIKV, and JEV in SD or DF to explore whether coinfection can lead to an increase in SD. After detection, six inpatients were infected with ZIKV in the SD group, and 0 were infected in the DF group. However, the clinical symptoms of these six coinfected inpatients were not obviously different from those of other SD patients. These results suggested that coinfection may not lead to an aggravation of the symptoms. Unfortunately, among the six ZIKV infected patients, the serum collection time of five of them is longer than 7 days, and the possibility of secondary dengue infection cannot be ruled out. Due to the small number of coinfections in this cohort, more patients needed to be enrolled, to study the role of ZIKV coinfection in SD patients.

The virulence of viruses could influence the occurrence of SD (Tuiskunen et al., 2011). In this study, we selected three DENV-2 epidemic strains from DF or DHF patients and performed whole genome sequencing and analysis. Compared with the sequences before 2019, the GC/AT in five of six strains increased in 2019. However, there was no regularities of the mutations between SD and DF sequences. The results showed that all the sequences from DF and SD belonged to cosmopolitan, and five of them were in a cluster.

The aim of this study was to investigate the causes of SD through the demographic information of SD patients, the co-infection of mosquito-borne viruses, the identification of DENV serotypes, the presence of DENV secondary infections, and the characteristics of the samples of the DENV complete genomes in Xishuangbanna, 2019 (Zhang et al., 2021). The prevalence of three dengue virus serotypes before 2019 might mediate subneutralization titer antibodies and lead to secondary infections, increasing the number of severe dengue patients in Xishuangbanna in 2019. The results of this study might provide insight into early prognostic factors associated with a severe disease progression and improve the rates of early diagnosis and successful treatment. The currently datas were very limited and only suggestive. More dengue patients should be recruited for those study. More other risk factors, especially environmental factors, the basic situation of patients should be included in those studys.

## Data Availability Statement

The datasets presented in this study can be found in online repositories. The names of the repository/repositories and accession number(s) can be found below: https://www.ncbi.nlm.nih.gov/genbank/, MZ452990-MZ453011.

## Ethics Statement

The studies involving human participants were reviewed and approved by the Ethics Committee of the Institute of Medical Biology, Chinese academy of Medical Sciences, China. Written informed consent to participate in this study was provided by the participants’ legal guardian/next of kin.

## Author Contributions

XW and QS have drafted and revised the manuscript. XW contributed to the sequencing, major experiment and analysis of data. QS and PL have designed and administered the study. TL, YS, JZ, XS, and DL contributed to sample collection. All other co-authors contributed to its finalization and approval for publication.

## Conflict of Interest

The authors declare that the research was conducted in the absence of any commercial or financial relationships that could be construed as a potential conflict of interest.

## Publisher’s Note

All claims expressed in this article are solely those of the authors and do not necessarily represent those of their affiliated organizations, or those of the publisher, the editors and the reviewers. Any product that may be evaluated in this article, or claim that may be made by its manufacturer, is not guaranteed or endorsed by the publisher.

## Footnotes

1
http://www.megasoftware.net/

## References

[B1] Bhatt P. Sasidharan S. P. Varma M. Arunkumar G. (2020). Current understanding of the pathogenesis of dengue virus infection. *Curr. Microbiol.* 78 17–32. 10.1007/s00284-020-02284-w 33231723 PMC7815537

[B2] Chaturvedi U. C. Agarwal R. Elbishbishi E. A. Mustafa A. S. (2000). Cytokine cascade in dengue hemorrhagic fever: implications for pathogenesis. *FEMS Immunol. Med. Microbiol.* 28 183–188. 10.1111/j.1574-695X.2000.tb01474.x 10865168

[B3] Cui X. G. Li L. H. Zhou H. N. Shan X. Y. Bai C. H. (2016). Clinical characteristics of 109 dengue virus type 2 infected cases in Xishuangbanna. *Chin. J. Infect. Dis.* 34 231–232.

[B4] Fried J. R. Gibbons R. V. Kalayanarooj S. Thomas S. J. Srikiatkhachorn A. Yoon I.-K. (2010). Serotype-specific differences in the risk of dengue hemorrhagic fever: an analysis of data collected in Bangkok, Thailand from 1994 to 2006. *PLoS Negl. Trop. Dis.* 4:e617. 10.1371/journal.pntd.0000617 20209155 PMC2830471

[B5] Guzman M. G. Harris E. (2015). Dengue. *Lancet* 385 453–465. 10.1016/S0140-6736(14)60572-9 25230594

[B6] Hawley W. A. Reiter P. Copeland R. S. Pumpuni C. B. Craig G. B. (1987). Aedes albopictus in North America: probable introduction in used tires from northern Asia. *Science* 236 1114–1116. 10.1126/science.3576225 3576225

[B7] Htun N. S. N. Odermatt P. Eze I. C. Boillat-Blanco N. D’Acremont V. Probst-Hensch N. (2015). Is diabetes a risk factor for a severe clinical presentation of dengue? – review and metaanalysis. *PLoS Negl. Trop. Dis.* 9:e0003741. 10.1371/journal.pntd.0003741 25909658 PMC4409149

[B8] Jiang L. M. Ma D. H. Ye C. Li L. Li X. Yang J. (2018). Molecular characterization of dengue virus serotype 2 cosmospolitan genotype from 2015 dengue outbreak in Yunnan, China. *Front. Cell. Infect. Microbiol.* 8:219. 10.3389/fcimb.2018.00219 29998087 PMC6030573

[B9] Liu Q. Y. (2020). Dengue fever in China: new epidemical trend, challengesand strategies for prevention and control. *Chin. J. Vector Biol. Control* 31 1–6.

[B10] Ma D. H. Shan X. Y. Li L. H. Li D. Ma D. Li T. (2016). An analysis of clinical features of dengue fever in the border areas of Xishuangbanna, China, Laos and Myanmar. *J. Trop. Dis. Parasitol.* 14 231–232.

[B11] Murphy B. R. Whitehead S. S. (2011). Immune response to dengue virus and prospects for a vaccine. *Annu. Rev. Immunol.* 29 587–619. 10.1146/annurev-immunol-031210-101315 21219187

[B12] Norshidah H. Vignesh R. Lai N. S. (2021). Updates on dengue vaccine and antiviral: where are we heading? *Molecules* 26, 6768. 10.3390/molecules26226768 34833860 PMC8620506

[B13] Sharp T. M. Tomashek K. M. Read J. S. Margolis H. S. Waterman S. H. (2017). A new look at an old disease: recent insights into the global epidemiology of dengue. *Curr. Epidemiol. Rep.* 4 11–21. 10.1007/s40471-017-0095-y 28251039 PMC5306284

[B14] Simmons C. P. Farrar J. J. Nguyen V. Wills B. (2012). Dengue. *N. Engl. J. Med.* 366 1423–1432. 10.1056/NEJMra1110265 22494122

[B15] St. John A. L. Abraham S. N. Gubler D. J. (2013). Barriers to preclinical investigations of anti-dengue immunity and dengue pathogenesis. *Nat. Rev. Microbiol.* 11 420–426. 10.1038/nrmicro3030 23652323

[B16] Tuiskunen A. Monteil V. Plumet S. Boubis L. Wahlström M. Duong V. (2011). Phenotypic and genotypic characterization of dengue virus isolates differentiates dengue fever and dengue hemorrhagic fever from dengue shock syndrome. *Arch. Virol.* 156, 2023–2032. 10.1007/s00705-011-1100-2 21922323

[B17] Wang J. Xie L. Xie X. L. Jiang J. Y. Yang B. Li C. M. (2016). Dengue vectors and the natural infection in border with Laos, Jiangcheng County, China. *Chin. J. Zoonoses* 32 843–849.

[B18] Wei H. Y. Shu P. Y. Hung M. N. (2016). Characteristics and risk factors for fatality in patients with dengue hemorrhagic fever, Taiwan, 2014. *Am. J. Trop. Med. Hyg.* 95 322–327. 10.4269/ajtmh.15-0905 27273649 PMC4973177

[B19] World Health Organization (2009). *Dengue Guidelines for Diagnosis, Treatment, Prevention and Control R.* Geneva: World Health Organization, 14. 23762963

[B20] Yue Y. J. Ren D. S. Liu X. B. Wu H. X. Guo Y. H. Zhou N. (2020). A study on spatial characteristics and correlations of different types of dengue cases in mainland China, 2014-2018. *Chin. J. Vector Biol. Control* 31 517–520.

[B21] Zhang H. L. (2021). Cross-border spread, indigenous transmission, development trend, and control strategy for dengue fever and chikungunya fever in Yunnan Province, China. *Chin. J. Vector Biol. Control* 32 12–18.

[B22] Zhang J. Shu Y. Shan X. Y. Li D. Ma D. Li T. (2021). Co-circulation of three dengue virus serotypes led to a severe dengue outbreak in Xishuangbanna, a border area of China, Myanmar, and Laos, in 2019. *Int. J. Infect. Dis.* 107 15–17. 10.1016/j.ijid.2021.04.010 33857610

