# Corrigendum: Clinical Characteristics and Risk Factors for Severe Dengue Fever in Xishuangbanna, During the Dengue Outbreak in 2019

**DOI:** 10.3389/fmicb.2022.939709

**Published:** 2022-06-20

**Authors:** Xiaodan Wang, Tingting Li, Yun Shu, Juan Zhang, Xiyun Shan, Daiying Li, Dehong Ma, Shuying Long, Yue Pan, Junying Chen, Pinghua Liu, Qiangming Sun

**Affiliations:** ^1^Institute of Medical Biology, Chinese Academy of Medical Sciences, Peking Union Medical College, Kunming, China; ^2^Yunnan Key Laboratory of Vaccine Research & Development on Severe Infectious Diseases, Kunming, China; ^3^Yunnan Key Laboratory of Vector-Borne Infectious Disease, Kunming, China; ^4^Xishuangbanna Dai Autonomous Prefecture People's Hospital, Jinhong, China; ^5^Kunming Medical University, Kunming, China

**Keywords:** severe dengue fever, IgG, IgM, dengue inpatients, dengue gene sequence

In the original article, the reference for “**The genome encodes a polyprotein, which is processed into three structural proteins [the capsid (C), premembrane (prM), and envelope (E) protein] and seven non-structural proteins (NS1-NS5) (Langerak et al., 2019)**” was incorrectly written as “Langerak, T., Mumtaz, N., Tolk, V. I., van Gorp, E. C. M., Martina, B. E., Rockx, B., et al. (2019). The possible role of cross-reactive dengue virus antibodies in Zika virus pathogenesis. *PLoS Pathog*. 15:e1007640. doi: 10.1371/journal.ppat. 1007640.” It should be “Guzman, M. G., and Harris, E. (2015). Dengue. *Lancet* 385, 453–465. doi: 10.1016/S0140-6736(14)60572-9.”

The reference for “**There are currently four circulating serotypes (DENV-1 to DENV-4) that exhibit up to 70% sequence homology (Matsumoto et al., 2011)**” was incorrectly written as “Matsumoto, M., Oshiumi, H., and Seya, T. (2011). Antiviral responses induced by the TLR3 pathway. *Rev. Med. Virol*. 21, 67–77. doi: 10.1002/rmv.680.” It should be “Bhatt, P., Sasidharan, S. P., Varma, M., and Arunkumar, G. (2020). Current understanding of the pathogenesis of dengue virus infection. *Curr. Microbiol*. 78, 17–32. doi: 10.1007/s00284-020-02284-w.”

The reference for “**Dengue was listed as a potential threat among ten diseases by the WHO in 2019 (Zhang et al., 2001)**” was incorrectly written as “Zhang, H. L., Zi, D. Y., Mi, Z. Q., and Gong, Z. D. (2001). Characterized distribution of Aedes albopictus and their relation with arbovirus in Yunnan province. *Chin. J. Vector Biol. Control* 12, 103–105.” It should be “Norshidah, H., Vignesh, R., and Lai, N. S. (2021). Updates on dengue vaccine and antiviral: where are we heading? *Molecules*. 26, 6768. doi: 10.3390/molecules26226768.”

The reference for “**The primers were as Supplementary Table 1 shown (Guzman and Harris**, **2015****)**” was incorrectly written as “Guzman, M. G., and Harris, E. (2015). Dengue. *Lancet* 385, 453–465.” It should be “Wang, J., Xie, L., Xie, X. L., Jiang, J. Y., Yang, B., Li, C. M., et al. (2016). Dengue vectors and the natural infection in border with Laos, Jiangcheng County, China. *Chin. J. Zoonoses* 32, 843–849.”

The reference for “**SD is currently believed to be mainly related to secondary heteromorphic DENV infection, coinfection of mosquito-borne viruses, viral variation and host immune response (Wei et al., 2016; Jiang et al., 2018; Tuiskunen et al.**, **2011****)**” was incorrectly written as “Wei, H. Y., Shu, P. Y., and Hung, M. N. (2016). Characteristics and risk factors for fatality in patients with dengue hemorrhagic fever, Taiwan, 2014. *Am. J. Trop. Med. Hyg*. 95, 322–327. doi: 10.4269/ajtmh.15-0905; Jiang, L. M., Ma, D. H., Ye, C., Li, L., Li, X., Yang, J., et al. (2018). Molecular characterization of dengue virus serotype 2 cosmospolitan genotype from 2015 dengue outbreak in Yunnan, China. *Front. Cell. Infect. Microbiol*. 8:219. doi: 10.3389/fcimb.2018.00219; Rathore, A. P. S., Farouk, F. S., and St. John, A. L. (2020). Risk factors and biomarkers of severe dengue. *Curr. Opin. Virol*. 43, 1–8. doi: 10.1016/j.coviro 2020.06.008.” It should be “St John, A. L., Abraham, S. N., and Gubler, D. J. (2013). Barriers to preclinical investigations of anti-dengue immunity and dengue pathogenesis. *Nat. Rev. Microbiol*. 11, 420–426. doi: 10.1038/nrmicro3030; Simmons, C. P., Farrar, J. J., Nguyen, V., and Wills, B. (2012). Dengue. *N. Engl. J. Med*. 366, 1423–1432. doi: 10.1056/NEJMra1110265.”

The reference for “**In South America, Southeast Asia and other countries, SD is considered to occur in children and infants (John et al., 2013)**” was incorrectly written as “John, A. L. S., Abraham, S. N., and Gubler, D. J. (2013). Barriers to preclinical investigations of anti-dengue immunity and dengue pathogene. *Nat. Rev. Microbiol*. 11(Suppl. 1) 420–426. doi: 10.1038/nrmicro3030.” It should be “Sharp, T. M., Tomashek, K. M., Read, J. S., Margolis, H. S., and Waterman, S. H. (2017). A new look at an old disease: recent insights into the global epidemiology of dengue. *Curr. Epidemiol. Rep*. 4, 11–21. doi: 10.1007/s40471- 017-0095-y.”

The reference for “**In 2019, there were 22599 cases of dengue fever, with an incidence rate of 1.63/10 million (Simmons et al.**, **2012****)**” was incorrectly written as “Simmons, C. P., Farrar, J. J., Nguyen, V., and Wills, B. (2012). Dengue. *N. Engl. J. Med*. 366, 1423–1432.” It should be “Liu Q. Y. (2020). Dengue fever in China: new epidemical trend, challenges and strategies for prevention and control. *Chin. J. Vector Biol. Control* 31, 1–6.”

The reference for “**According to former studies, DENV-2 and DENV-3 are more likely to cause SD than DENV-1 and DENV-4 (Sharp et al.**, **2017****)**” was incorrectly written as “Sharp, T. M., Tomashek, K. M., Read, J. S., Margolis, H. S., and Waterman, S. H. (2017). A new look at an old disease: recent insights into the global epidemiology of dengue. *Curr. Epidemiol. Rep*. 4, 11–21. doi: 10.1007/s40471-017-0095-y.” It should be “Fried, J. R., Gibbons, R. V., Kalayanarooj, S., Thomas, S. J., Srikiatkhachorn, A., Yoon, I.-K., et al. (2010). Serotype-specific differences in the risk of dengue hemorrhagic fever: an analysis of data collected in Bangkok, Thailand from 1994 to 2006. *PLoS Negl. Trop. Dis*. 4:e617. doi: 10.1371/journal.pntd.0000617.”

The reference for “**When the host is reinfected with heterotypic DENV, the E or PrM antibody produced by the first infection causes a subneutralization titer in the body and forms an immune complex with the virus being infected, increasing the infection rate and replication of the virus (Fried et al.**, **2010****)**” was incorrectly written as “Fried, J. R., Gibbons, R. V., Kalayanarooj, S., Thomas, S. J., Srikiatkhachorn, A., Yoon, I.-K., et al. (2010). Serotype-specific differences in the risk of dengue hemorrhagic fever: an analysis of data collected in Bangkok, Thailand from 1994 to 2006. *PLoS Negl. Trop. Dis*. 4:e617. doi: 10.1371/journal.pntd.0000617.” It should be “Murphy, B. R., and Whitehead, S. S. (2011). Immune response to dengue virus and prospects for a vaccine. *Annu. Rev. Immunol*. 29, 587–619. doi: 10.1146/ annurev-immunol-031210-101315.”

The reference for “**The virulence of viruses could influence the occurrence of SD (Murphy and Whitehead**, **2011****)**” was incorrectly written as “Murphy, B. R., and Whitehead, S. S. (2011). Immune response to dengue virus and prospects for a vaccine. *Annu. Rev. Immunol*. 29, 587–619. doi: 10.1146/ annurev-immunol-031210-101315.” It should be “Tuiskunen A, Monteil V, Plumet S, Boubis L, Wahlström M, Duong V, et al. Phenotypic and genotypic characterization of dengue virus isolates differentiates dengue fever and dengue hemorrhagic fever from dengue shock syndrome. *Arch Virol*. (2011) 156:2023–32. doi: 10.1007/s00705-011-1100-2.”

The authors apologize for those errors and state that those do not change the scientific conclusions of the article in any way. The original article has been updated.

## Publisher's Note

All claims expressed in this article are solely those of the authors and do not necessarily represent those of their affiliated organizations, or those of the publisher, the editors and the reviewers. Any product that may be evaluated in this article, or claim that may be made by its manufacturer, is not guaranteed or endorsed by the publisher.
